# Fluoroquinolones and the risk of panic attacks: a systematic review and disproportionality analysis using individual case safety reports from the FDA Adverse Event Reporting System (FAERS) database

**DOI:** 10.1093/jac/dkag083

**Published:** 2026-03-06

**Authors:** Keeirah Hiertika Raguram, Manroop Sidhu, Mohammad Ali Omrani, Bala Swetha Baskaran, Niaz Chalabianloo, Manik Chhabra, Hugues Sampasa-Kanyinga, Flory Tsobo Muanda

**Affiliations:** Department of Physiology and Pharmacology, Western University, London, Ontario, Canada; Department of Physiology and Pharmacology, Western University, London, Ontario, Canada; Department of Physiology and Pharmacology, Western University, London, Ontario, Canada; Department of Physiology and Pharmacology, Western University, London, Ontario, Canada; Department of Physiology and Pharmacology, Western University, London, Ontario, Canada; ICES Western, Kidney, Dialysis & Transplantation Research Program, London, Ontario, Canada; Department of Epidemiology and Biostatistics, Western University, London, Ontario, Canada; London Health Sciences Centre, Lawson Health Research Institute, London, Ontario, Canada; Healthy Active Living and Obesity Research Group, Children's Hospital of Eastern Ontario Research Institute, Ottawa, Ontario, Canada; Department of Physiology and Pharmacology, Western University, London, Ontario, Canada; ICES Western, Kidney, Dialysis & Transplantation Research Program, London, Ontario, Canada; Department of Epidemiology and Biostatistics, Western University, London, Ontario, Canada; London Health Sciences Centre, Lawson Health Research Institute, London, Ontario, Canada

## Abstract

**Background and objectives:**

Fluoroquinolones are linked with increased risk of CNS adverse events, such as anxiety and depression. Recently, case reports have linked fluoroquinolone use and panic attacks. However, current evidence exploring the link between fluoroquinolone use and panic attacks remains limited and requires investigation for safe use. To systematically review the literature on fluoroquinolone use and the risk of panic attacks, and to study this association by comparing fluoroquinolones with other antibiotics using the FDA Adverse Event Reporting System (FAERS) database.

**Methods:**

MEDLINE and Embase databases were searched to identify relevant studies for systematic review. Active-comparator restricted disproportionality analyses using FAERS (2004Q1-2024Q4) were performed for ciprofloxacin, levofloxacin, and moxifloxacin compared to azithromycin and trimethoprim/sulfamethoxazole. Reporting odds ratios (ROR), proportional reporting ratios, adjusted ROR for potential confounders, and Bayesian analyses were conducted to detect safety signals for MedDRA term ‘panic attack’.

**Results:**

The systematic review identified 12 studies (4 clinical trials, 8 publications describing 11 case reports), with the prevalence of panic attacks ranging between 0.46% and 1.76% in trials. Disproportionality analysis showed that, compared to azithromycin, fluoroquinolones were associated with a 6-fold increase in reports of panic attacks and a 12-fold increase compared to trimethoprim/sulfamethoxazole. Results were consistent across Bayesian analyses.

**Conclusion:**

The findings suggest an association between fluoroquinolones and increased risk of panic attacks, underscoring the need for validation through pharmacoepidemiological studies. Due to reliance on spontaneous reports, causal relationships cannot be determined for clinical recommendations. These results offer insights for research on CNS safety profiles of fluoroquinolones.

## Introduction

### Background

Fluoroquinolones (FQs) are among the most widely prescribed antibiotics, prized for their broad-spectrum activity and excellent oral bioavailability. They are commonly used as first-line therapy for bacterial infections of the respiratory, urinary, and gastrointestinal tracts.^1,2^ Despite their widespread use, FQs have been linked to serious adverse effects, including tendon rupture, hypoglycaemia, and increasingly, CNS-related events.^3,4^ Post-marketing surveillance has identified various neuropsychiatric risks associated with FQs—notably anxiety, insomnia, mood changes and psychosis. These concerns prompted several FDA safety communications, including a 2016 boxed warning specifically highlighting the risk of anxiety, delirium and other serious CNS adverse effects. FQs readily cross the blood–brain barrier due to their high lipophilicity, potentially disrupting excitatory and inhibitory CNS pathways.^5^ Case reports have frequently documented panic attacks—sudden, intense episodes of anxiety and distress—following fluoroquinolone use. Yet, no systematic review has specifically summarized the literature on fluoroquinolone-related panic attacks, and no large-scale pharmacovigilance studies using the FDA Adverse Event Reporting System (FAERS) have rigorously investigated this association. This knowledge gap limits clinicians’ ability to recognize and manage fluoroquinolone-related CNS risks.

### Objectives

We performed a systematic review of the literature and active-comparator disproportionality analyses (ACR-DA) using the FAERS database to evaluate the association between fluoroquinolone use and panic attacks. This dual approach allowed us to assess existing clinical evidence while further investigating the signal through pharmacovigilance data.

## Materials and methods

### Systematic review

#### Protocol registration

The systematic review was conducted according to the standards of the Preferred Reporting Items for Systematic Reviews and Meta-Analyses (PRISMA) Statement (Tables S1 and S2, available as Supplementary data at *JAC* Online).^6^ This systematic review was registered prospectively in the International Prospective Register of Systematic Reviews (PROSPERO) (Registration Identification: CRD42024613601, https://www.crd.york.ac.uk/PROSPERO/view/CRD42024613601). MEDLINE (via Ovid) and Embase (via Ovid) were systematically searched with the help of a health sciences librarian to identify relevant studies from inception and searches conducted up to 28 May 2025 without any language or date restrictions. This dual-database strategy was chosen because searching two or more databases provides over 95% coverage of relevant references.^7^ Additionally, the reference lists of included articles were examined to identify other potentially relevant studies. Search strings were utilized on MEDLINE and Embase databases, respectively, in Tables S3 and S4.

#### Inclusion and exclusion criteria

This systematic review was limited to studies conducted in human subjects of all age groups that reported fluoroquinolone use and panic attacks or acute anxiety as outcomes. Eligible study designs included clinical trials, observational studies, and case reports. Exclusion criteria included reviews and editorials, grey literature (e.g. institutional reports and unpublished theses), studies without full-text availability, and *in vitro* or non-human studies. To reduce potential confounding, studies involving patients with bipolar disorder or schizophrenia were excluded due to the higher baseline likelihood of panic attacks or acute anxiety in these populations.

#### Outcomes of interest

The outcomes of interest were panic attacks, which are defined as sudden peaks of intense anxiety, fear, or distress that escalate to peaks within minutes according to DSM-5.^8^ To ensure all potential cases of panic attacks were captured in this systematic review, articles that reported acute anxiety, which are similar surges of sudden distress, were also included. These adverse effects (AEs) were chosen for this review because panic attacks are frequently reported but underexplored potential CNS-related AEs linked with fluoroquinolone use.

#### Study selection and data extraction

References were imported into Covidence, a web-based application developed by Veritas Health Innovation (Melbourne, Australia). Covidence streamlines systematic reviews by supporting study screening, full-text assessment, and data extraction, and allows effective collaboration and documentation among reviewers.^9^

After the removal of duplicates automatically by Covidence, two independent reviews (K.R. and M.S.) screened titles and abstracts, followed by full text using a priori inclusion and exclusion criteria. Any disagreements were resolved by consensus or, when necessary, through another reviewer (F.M.).

Using Microsoft Excel, data extraction for each of the studies included in this systematic review was standardized to ensure consistency. In the standardized form, the first author, year of publication, study design, location, patient characteristics and demographics (e.g. age and sex), specifics of fluoroquinolone exposure (e.g. duration and dosage of treatment), clinical indications, time to panic attack or acute anxiety onset, and anxiety-related outcomes were extracted.

#### Risk of bias assessment

For each study included in this systematic review, two independent reviewers (K.R. and M.S.) conducted a Risk of Bias 2 (ROB 2) assessment to evaluate the methodological quality of the included studies. The Naranjo scale was applied to case reports to examine the likelihood of panic attacks or acute anxiety being associated with fluoroquinolone use.^10^ The Risk of Bias 2 (ROB2) tool was used to assess the quality of the clinical trials, such that each clinical trial was classified as either low risk, some concerns, or high risk.^11^

#### Data synthesis

Descriptive analyses were performed for each study included in this systematic review. In brief, the features of each study were gathered when reported. The study design was recorded, and objectives and collection techniques were included. For each study, population characteristics were summarized, including demographics and health status. The components of intervention were documented, including duration, frequency routes of administration of FQs. Additionally, the primary outcome of each study was collected. In clinical trials, effect estimates [risk ratio (RR) with 95% confidence interval (CI)] were calculated if data were available for FQs and comparator arms. The RR is the ratio of the cumulative incidence of panic attacks in the fluoroquinolone group compared to the control group.^12^

### Active-comparator restricted disproportionality analysis

An ACR-DA was conducted using Individual Case Safety Reports (ICSRs) from the FAERS database to more accurately quantify disproportionate reporting of AEs.^13,14^ The ACR-DA method compared the reporting rates of potential adverse drug reactions for the study medication, FQs, with the reporting rates of the same reactions for active comparator medications used for a similar clinical indication.^15^ This ACR-DA follows the Reporting of a Disproportionality Analysis for Drug Safety Signal Detection Using Individual Case Safety Reports in PharmacoVigilance guidelines (Tables S5 and S6).^16^ The focused approach of ACR-DA reduced the occurrence of false positive safety signals from disproportionate reporting in the FAERS database.^15^ The reporting odds for panic attacks in ICSRs for FQs—including ciprofloxacin, levofloxacin and moxifloxacin—were compared to the reporting odds for panic attacks for a group of active comparator medications: the non-fluoroquinolone antibiotics azithromycin and trimethoprim/sulfamethoxazole.

#### Data sources

The FAERS is a publicly available database maintained by the US Food and Drug Administration that collects reports of adverse drug events and medication errors. Reporting to FAERS is mandatory for pharmaceutical manufacturers and voluntary for healthcare professionals and patients, with submissions typically made using standardized MedWatch forms or electronic reporting systems.^17^

The FAERS database was used to collect reports of panic attacks following fluoroquinolone or non-fluoroquinolone antibiotic use through the OpenFDA platform, which is publicly available. The OpenFDA reduced duplicate reports and was more user-friendly compared to the raw FAERS data.^17^ Using the MedDRA term ‘panic attacks’, the reporting of panic attacks was identified on the FAERS database from the first quarter of 2004 to the fourth quarter of 2024. Related terms such as ‘panic reaction’ were not included to maintain the specificity of the main outcome of interest.

#### Data cleaning

Duplicate cases were identified and removed using the unique case identifier provided in OpenFDA.^18^ Adverse events were analysed at the individual report level, without deduplication of outcomes across reports, which is consistent with standard disproportionality analysis practices. Only cases in which the medication was classified as the primary suspect were included. The outcome of interest was restricted to the MedDRA preferred term (PT) ‘Panic attack’; related terms (e.g. ‘Panic reaction’) were not included to maintain outcome specificity. Extensive data cleaning was performed due to inconsistencies in drug names, particularly in the ‘medicinal product’ field. Drug names were normalized using regular expressions and mapped to standardized vocabularies, including RxNorm and the Observational Health Data Sciences and Informatics framework.^18^ RxNorm Concept Unique Identifiers, generic names, and Anatomical Therapeutic Chemical classification codes were added to enrich the dataset.^19^ Drug role fields in OpenFDA were mapped to FAERS role codes to facilitate identification of primary suspect drugs. Clinical characteristics extracted from individual case safety reports included age, sex, time to panic attack, indication for use, reporting country and serious outcomes.

#### Exposed and active-comparator groups

The exposed cohort consisted of ICSRs in which FQs—ciprofloxacin, levofloxacin and moxifloxacin—were identified as the primary suspect for the adverse event. Two comparator groups were defined: azithromycin and trimethoprim/sulfamethoxazole. These agents were selected because they share overlapping clinical indications with FQs for the treatment of respiratory and urinary tract infections, thereby minimizing confounding by indication. Both antibiotics exhibit some degree of central nervous system penetration, although to a lesser extent than FQs (Table S7). To further account for potential differences in blood–brain barrier permeability, a within-class analysis among FQs was conducted to assess whether specific agents were associated with higher reporting rates of panic attacks.

#### Statistical analysis

All analyses were exploratory and hypothesis-generating. Disproportionality analyses were conducted using both frequentist measures, including the proportional reporting ratio (PRR) and reporting odds ratio (ROR), and Bayesian measures, such as the information component (IC). The ROR quantifies the strength of association between an exposure and an outcome, while the PRR assesses the frequency of adverse events associated with the study medication compared to other drugs in the database.^20,21^ Logistic regression analyses were used to calculate adjusted RORs, accounting for potential confounders, including age, sex and indications of respiratory and urinary tract infections. Since the FAERS database does not contain denominator data on drug exposure, adjusted RORs represent relative reporting rates rather than absolute incidence or risk. A safety signal was defined according to three criteria: IC025 (the lower 2.5% confidence limit of the IC) >0, the lower bound of the 95% CI of the ROR >1.0, and a minimum of three reported cases.^21^ Forest plots were generated to visualize comparisons between FQs and the active comparators. All statistical analyses were performed using SAS (Version 8.3, SAS Studio, Cary, NC, USA) and R (Version 4.3.3).

## Results

### Systematic review

#### Study selection

**Figure 1.**
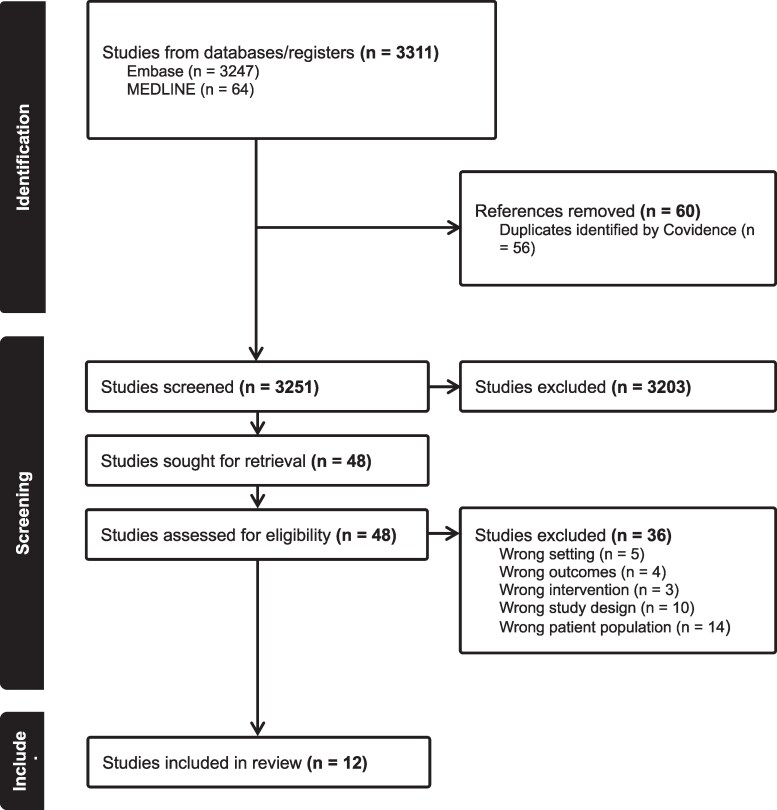
Preferred Reporting Items for Systematic Reviews and Meta-Analyses (PRISMA) flow diagram of study selection for the systematic review. Preferred Reporting Items for Systematic Reviews and Meta-Analyses (PRISMA) flow diagram illustrating the identification (*n* = 3311), screening (*n* = 3251), and inclusion of eligible studies (*n* = 12) in the systematic review from the databases Embase and MEDLINE. Studies were included if the study design was clinical trials, observational studies, or case reports; the intervention was fluoroquinolones; the comparator was non-fluoroquinolone antibiotics, no treatment, or placebo; the outcome was panic attacks or acute anxiety. (Indexed 1946 to 28 May 2025).

Of 3311 identified citations, 12 unique studies were included: 4 clinical trials, and 8 publications describing case reports. Figure 1 displays the PRISMA flow diagram, which describes complete process of study selection. Among these, two publications were case series (describing two and three cases, respectively), while the remaining six were individual case reports. Consequently, a total of 11 individual cases were identified involving the use of FQs associated with panic attacks or acute anxiety.

##### Clinical trials

Table S8 summarizes the included clinical trials. The prevalence of panic attacks or acute anxiety was low, ranging from 0.5% to 1.8%, with wide confidence intervals. In Brazil, a multicentre trial by Medeiros *et al*.^22^ reported 23 cases (0.5%) of CNS alterations, including anxiety, among 5044 patients receiving gatifloxacin. In Italy, Periti *et al*.^23^ observed one anxiety episode (0.7%) among 153 patients treated with intravenous ciprofloxacin. Only one trial, by Petitpretz *et al*.^24^, included a direct comparator. Among patients with acute exacerbation of chronic obstructive bronchitis, levofloxacin (*n* = 340) was associated with six panic attacks (1.8%), whereas cefuroxime (*n* = 349) had two cases (0.6%), resulting in a crude risk ratio of 3.08 (95% CI, 0.63–15.15). In the USA, Tack *et al*.^25^ reported one anxiety case (1.3%) among 76 patients receiving clinafloxacin.

Risk of bias assessment using ROB2 showed that three trials were high risk, while Petitpretz *et al*. had some concerns (Table S9).

##### Case reports

The eight publications describing case reports included 11 cases (Table S10), with nine (81.8%) reporting acute anxiety and three (27.3%) reporting panic attacks associated with fluoroquinolone use.^26–33^ Overall, clinical presentations in these cases were limited to panic attacks and acute anxiety; notably, no other neuropsychiatric disturbances, such as hallucinations or agitation, were observed. The median age was 32 years (IQR: 25–40), and five patients (45.5%) were male. Indications for fluoroquinolone use included lower respiratory tract infection (36.4%), pelvic inflammatory disease (18.2%), sinusitis (18.2%), indwelling bile duct stent with cholangitis (9.1%), bilateral submandibular abscesses (9.1%) and acute febrile upper airway infection (9.1%). The most frequently prescribed FQs were levofloxacin (45.5%), moxifloxacin (36.4%) and ofloxacin (18.2%). The median time to onset of panic attacks or acute anxiety was 48 h (IQR: 5–72). All but one patient (9.1%) discontinued fluoroquinolone treatment; in one case, a 25-year-old woman continued treatment and returned to normal 24 h after completing antibiotics. For the others, the median time to symptom relief after discontinuation was 1.5 days (IQR: 1–5.5). Using the Naranjo scale, 10 of 11 cases were rated as probable (scores 5–8), and one case was possible (score 3) (Table S11).

### Active-comparator restricted disproportionality analysis

#### General characteristics

After deduplication, 114 130 ICSRs were identified in FAERS where FQs (ciprofloxacin, levofloxacin and moxifloxacin) and comparator antibiotics (azithromycin, trimethoprim/sulfamethoxazole) were the primary suspect drugs (Figure S1). In all subsequent calculations, the denominator is defined as the total number of ICSRs for each respective drug. Most ICSRs involved ciprofloxacin (*n* = 33 661; 29.5%), levofloxacin (*n* = 32 873; 28.8%) and moxifloxacin (*n* = 16 098; 14.1%) (Table 1). Panic attacks were reported in 1072 ICSRs (0.9%), distributed as ciprofloxacin (*n* = 609; 56.8%), levofloxacin (*n* = 305; 28.5%), moxifloxacin (*n* = 108; 10.1%), azithromycin (*n* = 40; 3.7%) and trimethoprim/sulfamethoxazole (*n* = 10; 0.9%). The median age of patients with fluoroquinolone-related panic attacks ranged from 39 to 40 years, and females accounted for 56%–74% of cases. Median onset of panic attacks was shortest for moxifloxacin (1.5 days, IQR 0.5–6), followed by ciprofloxacin and levofloxacin (5 days, IQR 2–9). Serious outcomes included disabling events, hospitalization and life-threatening events. Disabling was most frequent for ciprofloxacin (*n* = 105; 17.2%) and levofloxacin (*n* = 42; 13.8%), while hospitalization predominated for moxifloxacin (*n* = 10; 9.3%) and the non-fluoroquinolone antibiotics. Most reports originated from the USA, ranging from 35% (*n* = 214/609) for ciprofloxacin to 90% (*n* = 9/10) for trimethoprim/sulfamethoxazole.

**Table 1.** Clinical characteristics of reports associated with all fluoroquinolones, ciprofloxacin, levofloxacin, moxifloxacin, azithromycin and trimethoprim/sulfamethoxazole-related panic attacks from the FDA Adverse Event Reporting System (FAERS) database (2004Q1 to 2024Q4)

	All fluoroquinolones	Ciprofloxacin	Levofloxacin	Moxifloxacin	Azithromycin	Trimethoprim/sulfamethoxazole
Total reports	82632	33661	32873	16098	21431	10067
Panic attack reports	1022 (1.2%)	609 (1.8%)	305 (0.9%)	108 (0.7%)	40 (0.2%)	10 (0.1%)
Age (year)						
Median age (IQR)	40.00 (32 to 50)	40 (32 to 49)	40 (34 to 50)	39 (32 to 52)	36.50 (29.5 to 43)	42.00 (32.5 to 44)
Age, No. (%)						
<18	0 (0)	7 (1.1%)	0 (0)	0 (0)	2 (5.0%)	0 (0)
18–<41	464 (45.4%)	287 (47.1%)	124 (40.7%)	46 (42.6%)	18 (45.0%)	4 (40.0%)
41–<57	238 (23.3%)	158 (25.9%)	58 (19.0%)	22 (20.4%)	7 (17.5%)	4 (40.0%)
57–<71	87 (8.5%)	49 (8.0%)	29 (9.5%)	9 (8.3%)	1 (2.5%)	0 (0)
≥71	34 (3.3%)	28 (4.6%)	2 (0.7%)	4 (3.7%)	0 (0)	0 (0)
Unknown	199 (19.5%)	80 (13.1%)	92 (30.2%)	27 (25.0%)	12 (30.0%)	2 (20.0%)
Sex						
Male	350 (34.3%)	227 (37.3%)	99 (32.5%)	24 (22.2%)	19 (47.5%)	3 (30.0%)
Female	613 (60.0%)	361 (59.3%)	172 (56.4%)	80 (74.1%)	19 (47.5%)	6 (60.0%)
Unknown	59 (5.8%)	21 (3.5%)	34 (11.2%)	4 (3.7%)	2 (5.0%)	1 (10.0%)
Weight						
Median weight (IQR)	70.48 (60 to 81.8)	70 (60 to 81)	72 (58 to 84.4)	68.04 (59.0 to 86.0)	62.59 (55.8 to 68.0)	62.60 (56.3 to 72.1)
<43	2 (0.2%)	2 (0.3%)	0 (0)	0 (0)	2 (5.0%)	1 (10.0%)
43–<76	500 (48.9%)	318 (52.2%)	145 (47.5%)	37 (34.3%)	18 (45.0%)	6 (60.0%)
76–<92	175 (17.1%)	107 (17.6%)	56 (18.4%)	12 (11.1%)	3 (7.5%)	1 (10.0%)
≥92	103 (10.1%)	56 (9.2%)	37 (12.1%)	10 (9.3%)	2 (5.0%)	1 (10.0%)
Unknown	242 (23.7%)	126 (20.7%)	67 (22.0%)	49 (45.4%)	15 (37.5%)	0 (0)
Time to panic attack reports						
Median time to panic attack reports (IQR), days	4.00 (2 to 9)	5 (2 to 9)	5 (2 to 9)	1.50 (0.5 to 6.0)	2.00 (0 to 4.0)	4.00 (3 to 6)
<2	133 (13.0%)	69 (11.3%)	36 (11.8%)	28 (25.9%)	8 (20.0%)	1 (10.0%)
2–<5	154 (15.1%)	99 (16.3%)	45 (14.8%)	10 (9.3%)	11 (27.5%)	4 (40.0%)
5–<10	173 (16.9%)	106 (17.4%)	55 (18.0%)	12 (11.1%)	2 (5.0%)	2 (20.0%)
10–<20	76 (7.4%)	44 (7.2%)	27 (8.9%)	5 (4.6%)	2 (5.0%)	2 (20.0%)
≥20	34 (3.3%)	18 (3.0%)	15 (4.9%)	1 (0.9%)	0 (0)	0 (0)
Unknown	452 (44.2%)	273 (44.8%)	127 (41.6%)	52 (48.2%)	17 (42.5%)	1 (10.0%)
Indication of use						
Urinary tract infection	308 (30.1%)	259 (42.5%)	41 (13.4%)	8 (7.4%)	1 (2.5%)	1 (10.0%)
Respiratory tract infection	190 (18.6%)	32 (5.3%)	102 (33.4%)	56 (51.9%)	9 (22.5%)	3 (30.0%)
Other	261 (25.5%)	261 (42.9%)	0 (0)	0 (0)	25 (62.5%)	4 (40.0%)
Unknown	263 (25.7%)	57 (9.4%)	162 (53.1%)	44 (40.7%)	5 (12.5%)	2 (20.0%)
Serious outcomes						
Death	3 (0.3%)	0 (0)	1 (0.3%)	2 (1.9%)	0 (0)	0 (0)
Disabling	152 (14.9%)	105 (17.2%)	42 (13.8%)	5 (4.6%)	2 (5.0%)	0 (0)
Hospitalization	72 (7.1%)	39 (6.4%)	23 (7.5%)	10 (9.3%)	3 (7.5%)	1 (10.0%)
Life-threatening	45 (4.4%)	27 (4.4%)	13 (4.3%)	5 (4.6%)	2 (5.0%)	0 (0)
Not serious	71 (7.0%)	21 (3.5%)	28 (9.2%)	22 (20.4%)	2 (5.0%)	3 (30.0%)
Other	679 (66.4%)	417 (68.5%)	198 (64.9%)	64 (59.3%)	31 (77.5%)	6 (60.0%)
Report country						
USA	466 (45.6%)	214 (35.1%)	178 (58.4%)	74 (68.5%)	22 (55.0%)	9 (90.0%)
Great Britain	146 (14.3%)	137 (22.5%)	7 (2.3%)	2 (1.9%)	7 (17.5%)	0 (0)
Germany	244 (23.9%)	138 (22.7%)	97 (31.8%)	9 (8.3%)	0 (0)	0 (0)
Canada	34 (3.3%)	27 (4.4%)	0 (0)	7 (6.5%)	1 (2.5%)	0 (0)
Country not specified	3 (0.3%)	1 (0.2%)	1 (0.3%)	1 (0.9%)	0 (0)	0 (0)
Other	111 (10.9%)	86 (14.1%)	14 (4.6%)	11 (10.2%)	8 (20.0%)	0 (0)
Unknown	18 (1.8%)	6 (1.0%)	8 (2.6%)	4 (3.7%)	2 (5.0%)	1 (10.0%)

Total reports for each drug are indicated (All fluoroquinolones: *n* = 82 632; ciprofloxacin: *n* = 33 661; levofloxacin: *n* = 32 873; moxifloxacin: *n* = 16 098; azithromycin: *n* = 21 431; trimethoprim/sulfamethoxazole: *n* = 10 067). Panic attack reports are shown as *n* (%) for each drug.

Abbreviation: IQR, interquartile range.

#### Safety signal detection

**Figure 2.**
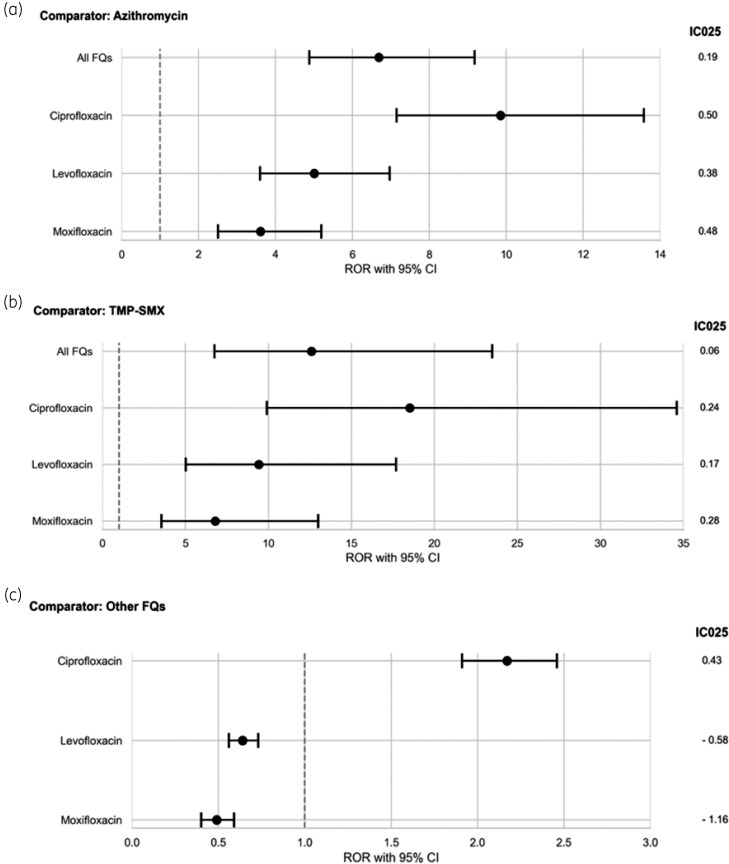
Forest plots for the active-comparator restricted disproportionality analysis (ACR-DA) of panic attacks for all fluoroquinolones (FQs), ciprofloxacin, levofloxacin and moxifloxacin compared to azithromycin, trimethoprim/sulfamethoxazole (TMP-SMX) or other fluoroquinolones. Forest plots show reporting odds ratios (RORs) and information components (IC025) for panic attack reports. Sample sizes (*n*) indicate the number of individual case safety reports (ICSRs) of panic attacks included in the analysis: All fluoroquinolones (1022/82 632, 1.2%), ciprofloxacin (609/33 661, 1.8%), levofloxacin (305/32 873, 0.9%), moxifloxacin (108/16 098, 0.7%), azithromycin (40/21 431, 0.2%), trimethoprim/sulfamethoxazole (10/10 067, 0.1%). Comparisons are shown in (a) azithromycin, (b) trimethoprim/sulfamethoxazole (TMP-SMX), (c) other fluoroquinolones (FQs).

A safety signal for panic attacks was identified for FQs compared to azithromycin and trimethoprim/sulfamethoxazole. Overall, FQs showed a 7-fold increase in panic attack reports versus azithromycin [1022/82 632 (1.2%) versus 40/21 431 (0.2%); ROR 6.69, 95% CI 4.88–9.18; IC025 0.19], which remained significant after adjusting for age, sex and infection type (aROR 5.73, 95% CI 4.16–7.88) (Table 2, Figure 2). Compared to trimethoprim/sulfamethoxazole, FQs exhibited a 13-fold increase in reports [1022/82 632 (1.2%) versus 10/10 067 (0.1%); ROR 12.59, 95% CI 6.75–23.48; IC025 0.06] and a 12-fold increase after adjustment (aROR 12.20, 95% CI 6.54–22.75) (Figure 2). Specifically, ciprofloxacin demonstrated the highest disproportionality. Compared to azithromycin, ciprofloxacin had a 10-fold increase in panic attack reports [609/33 661 (1.8%) versus 40/21 431 (0.2%); ROR 9.85, 95% CI 7.15–13.58; IC025 0.50] and 9-fold after adjustment (aROR 8.68, 95% CI 6.23–12.10). Versus trimethoprim/sulfamethoxazole, ciprofloxacin showed a 19-fold increase [609/33 661 (1.8%) versus 10/10 067 (0.1%); ROR 18.51, 95% CI 9.91–34.59; IC025 0.24] and 17-fold after adjustment (aROR 16.58, 95% CI 8.87–31.02) (Table 2, Figure 2). Levofloxacin also showed disproportionality compared to azithromycin (305/32 873 [0.9%] versus 40/21 431 [0.2%]; ROR 5.01, 95% CI 3.60–6.97; IC025 0.38) and trimethoprim/sulfamethoxazole [305/32 873 (0.9%) versus 10/10 067 (0.1%); ROR 9.42, 95% CI 5.02–17.69; IC025 0.17] (Table 2). Moxifloxacin showed similar trends [108/16 098 (0.7%) versus 40/21 431 (0.2%); ROR 3.61, 95% CI 2.51–5.19; IC025 0.48; versus trimethoprim/sulfamethoxazole: 108/16 098 (0.7%) versus 10/10 067 (0.1%); ROR 6.79, 95% CI 3.55–12.99; IC025 0.28] (Table 2). When FQs were compared within class, only ciprofloxacin showed a significantly higher number of panic attack reports, with a 2-fold increase compared to non-ciprofloxacin FQs [609/33 661 (1.8%) versus 413/48 971 (0.8%); ROR 2.17, 95% CI 1.91–2.46; IC025 0.43] (Table 2, Figure 2).

**Table 2.** ROR, aROR, PRR with 95% CI, IC and IC025 of panic attack reports for fluoroquinolones in the active-comparator restricted disproportionality analysis using data from the FDA Adverse Event Reporting System (FAERS)

Drug of interest	Comparators	ROR (95% CI)	aROR (95% CI)*^a^*	PRR (95% CI)	IC (IC_025_)
All fluoroquinolones*^b^*	Azithromycin	6.69 (4.88–9.18)	5.73 (4.16–7.88)	6.63 (4.83–9.09)	0.28 (0.19)
	Trimethoprim/sulfamethoxazole	12.59 (6.75–23.48)	12.20 (6.54–22.75)	12.45 (6.68–23.20)	0.15 (0.06)
Ciprofloxacin	Azithromycin	9.85 (7.15–13.58)	8.68 (6.23–12.10)	9.69 (7.04–13.34)	0.62 (0.50)
	Trimethoprim/sulfamethoxazole	18.51 (9.91–34.59)	16.58 (8.87–31.02)	18.21 (9.75–34.01)	0.35 (0.24)
	Other fluoroquinolones	2.17 (1.91–2.46)	2.07 (1.79–2.39)	2.15 (1.89–2.43)	0.55 (0.43)
Levofloxacin	Azithromycin	5.01 (3.60–6.97)	4.88 (3.49–6.82)	4.97 (3.58–6.91)	0.55 (0.38)
	Trimethoprim/sulfamethoxazole	9.42 (5.02–17.69)	8.92 (4.73–16.82)	9.34 (4.98–17.53)	0.34 (0.17)
	Other fluoroquinolones	0.64 (0.56–0.73)	0.72 (0.63–0.83)	0.64 (0.56–0.74)	−0.41 (−0.58)
Moxifloxacin	Azithromycin	3.61 (2.51–5.19)	3.08 (2.13–4.47)	3.59 (2.50–5.16)	0.76 (0.48)
	Trimethoprim/sulfamethoxazole	6.79 (3.55–12.99)	7.25 (3.47–15.12)	6.75 (3.54–12.90)	0.57 (0.28)
	Other fluoroquinolones	0.49 (0.40–0.59)	0.52 (0.42–0.64)	0.49 (0.40–0.60)	−0.88 (−1.16)

Total panic attacks reports for each drug are indicated: all FQs (1022/82,632, 1.2%), ciprofloxacin (609/33 661, 1.8%), levofloxacin (305/32 873, 0.9%), moxifloxacin (108/16 098, 0.7%), azithromycin (40/21 431, 0.2%) and trimethoprim/sulfamethoxazole (10/10 067, 0.1%).

Abbreviations: aROR, adjusted reporting odds ratio; CI, confidence interval; IC, information component; PRR, proportional reporting ratio; ROR, reporting odds ratio; IC_025_, lower bound of the 95% credibility interval for information component.

^a^Adjusted by age, sex, urinary tract infection indication, and respiratory tract infection indication.

^b^Panic attack reports associated with ciprofloxacin, levofloxacin and moxifloxacin.

## Discussion

### Summary

FQs are widely prescribed for respiratory and urinary tract infections and have been linked to various neuropsychiatric adverse effects, including panic attacks and anxiety-related AEs. However, their specific association with panic attacks has not been systematically studied. This study combined a systematic review of the literature and pharmacovigilance analyses using FAERS to evaluate whether FQs are disproportionately associated with reported panic attacks.

### Systematic review

The systematic review included 12 studies (4 clinical trials and 8 publications describing 11 case reports). Reported prevalence of panic attacks in clinical trials ranged from 0.5% to 1.8% with wide confidence intervals. Case reports generally suggested a probable causal link, supporting the hypothesis that FQs can trigger panic attacks in susceptible individuals.

### Active-comparator restricted disproportionality analysis

FAERS analyses revealed 1022 (1.2%) ICSRs reporting panic attacks among all fluoroquinolone-related reports. Disproportionality analyses demonstrated 6- to 12-fold higher reporting of panic attacks for FQs compared to non-fluoroquinolone antibiotics (azithromycin and trimethoprim/sulfamethoxazole). Although the observed RORs appear large, these estimates represent disproportionality signals reflecting relative reporting rates, rather than causal effects.

Ciprofloxacin showed the strongest signal, including a 2-fold increase compared to other FQs, suggesting that certain agents within the class may pose a higher neuropsychiatric risk. These findings were consistent across adjusted analyses and Bayesian measures (IC025).

### Comparison with other studies

The findings from this study align with previous studies reporting neuropsychiatric disorders associated with FQs.^3,34^ The observed frequency of panic attack reports among the clinical trials in this systematic review is consistent with the review conducted by Xie *et al*.^35^, who report that the incidence of neuropsychiatric events, such as confusion, restlessness, and suicidal depression, after the use of FQs, ranges from 1.0% to 4.4%. Moreover, the results from the case reports in this systematic review align with previous studies conducted on the existing literature, suggesting that FQs are known to be associated with neuropsychiatric adverse effects, including anxiety, hallucinations and confusion.^34,36^ However, no studies to date have specifically focused on the use of FQs and panic attacks, and our analysis provides a more detailed understanding of this potential association. Our findings are further contextualized by recent large-scale pharmacovigilance data from Wei et al., who used the FAERS database to characterize safety profiles of various FQs.^37^ While their study highlighted general class-level safety signals, it did not specifically examine neuropsychiatric outcomes such as panic attacks. By focusing on panic attacks, our study provides novel, granular insights into CNS-related risks and complements the broader pharmacovigilance evidence. The safety signals in our study were consistent with the ACR-DA conducted by Omrani *et al*.,^38^ in which potential safety signals for nightmare reports related to all FQs were indicated, particularly ciprofloxacin when compared to other FQs (ROR 1.22, 95% CI [1.04 to 1.44], IC025 0.17). This agrees with the results from this study and supports that ciprofloxacin warrants further caution due to the increased neuropsychiatric effects, such as panic attack reports and nightmares, compared not only to non-fluoroquinolone antibiotics but also to other fluoroquinolone antibiotics. While these findings from spontaneous reporting databases are hypothesis-generating, they highlight panic attacks as a distinct clinical phenotype that warrants further investigation using population-level longitudinal data. The mechanism underlying fluoroquinolone-associated panic attacks is not fully understood but is likely multifactorial. FQs readily penetrate the CNS due to their high lipophilicity and may act as GABA-A receptor antagonists, reducing inhibitory neurotransmission and increasing neuronal excitability, which can manifest as anxiety and panic symptoms.^34,35,39–41^ Additionally, FQs have been reported to induce mitochondrial toxicity, impairing cellular energy metabolism and generating oxidative stress, which may further contribute to CNS hyperexcitability and neuropsychiatric manifestations. Variability in patient susceptibility, including pre-existing neuropsychiatric conditions or genetic polymorphisms affecting neurotransmitter systems, may explain the heterogeneous clinical presentation observed in fluoroquinolone-exposed individuals.^34,36,42^

### Strengths and limitations

#### Novelty

This study has several strengths and provides a focused evaluation of FQs and panic attack reports. Our study provides the most comprehensive evaluation to date of the potential association between FQs and reported panic attacks, informing safer prescribing practices and guiding future research priorities. To date, it represents the most comprehensive evidence on the risk of panic attacks associated with FQs. Previous pharmacovigilance studies have reported associations between FQs and psychiatric adverse events, typically using broad outcomes or higher-level MedDRA categories.^3,35,38^ In contrast, this study specifically examined ‘panic attack’, a distinct clinical phenomenon characterized by sudden episodes of intense anxiety according to DSM-5.^8^ By analysing this PT alone, we aimed to determine whether FQs are disproportionately associated with panic attack reports. Additionally, we extended prior work through a systematic literature review and an ACR-DA, providing a more focused and methodologically robust assessment of this specific adverse event.

The systematic review identified relevant studies, highlighting that panic attacks are often under-recognized but clinically significant AEs. Using ACR-DA, we identified potential safety signals for all FQs from FAERS, a real-world pharmacovigilance database including reports from patients, healthcare professionals, and manufacturers. However, FAERS mainly captures reports from North America, primarily the United States, with a few reports from other regions. Therefore, the geographic distribution may not reflect global patterns of drug use. In addition, a higher proportion of reports involved female patients, which could reflect biological differences or reporting bias.

Active comparators (azithromycin and trimethoprim/sulfamethoxazole) mitigated potential confounding by respiratory and urinary tract infections, and an attempt was made to adjust for patient age, sex and infection indications.

The systematic review had limitations, including only 12 studies, eight of which were publications describing case reports, limiting generalizability. Since our study focused specifically on the clinical entities of panic attacks and acute anxiety, our search strategy prioritized specificity. However, this approach may have limited the retrieval of larger observational or pharmacovigilance studies in which such events are coded or reported under broader terms, such as ‘acute stress reactions’ or ‘anxiety-related symptoms’. To ensure transparency and facilitate further investigation, we have included the full search strategy in the Supplementary data (Tables S3 and S4). Three of four clinical studies had a high risk of bias due to deviations from intended interventions, and reporting of panic attacks was limited as many trials focused primarily on fluoroquinolone safety and efficacy.

The ACR-DA also has limitations. FAERS is prone to underreporting and bias, and ICSRs often lack detailed patient information and key confounders such as medical history. In addition, limited dose information and low counts of ofloxacin and norfloxacin led to their exclusion from the analysis. Masking bias can obscure signals when multiple drugs are reported.^43^ Further, the FAERS database is subject to notoriety bias, whereby reporting frequency can increase following regulatory warnings, such as the FDA boxed warnings in 2016 linking FQs to various mental health adverse events.^44^ This stimulated reporting may have inflated the number of reports independent of true event rates. Consequently, the observed disproportionalities may reflect, at least in part, stimulated reporting rather than a true association. While the ACR-DA can indicate potential safety signals, causal relationships cannot be established.^45^

### Conclusions

These findings identify a pharmacovigilance signal suggesting that FQs, particularly ciprofloxacin, may be associated with reports of panic attacks. Confirmation through controlled pharmacoepidemiologic studies is warranted.

## Supplementary Material

dkag083_Supplementary_Data

## Data Availability

The data that support the findings of this study are publicly available at https://open.fda.gov.

## Supplementary data

Figure S1 and Tables S1 to S11 are available as Supplementary data at *JAC* Online.
