# Supplementary material for: Fluoroquinolones and the risk of panic attacks: a systematic review and disproportionality analysis using individual case safety reports from the FDA Adverse Event Reporting System (FAERS) database
Source: J Antimicrob Chemother. 2026 Mar 6;81(4):dkag083. doi: 10.1093/jac/dkag083 (PMC13017743; doi:10.1093/jac/dkag083)
Supplement: dkag083_Supplementary_Data [file dkag083_supplementary_data.docx]

**Fluoroquinolones and the risk of panic attacks: A systematic review and disproportionality analysis using individual case safety reports from the FDA Adverse Event Reporting System (FAERS) database**

Keeirah Hiertika Raguram^1^, Manroop Sidhu^1^, Mohammad Ali Omrani^1^_,_ Bala Swetha Baskaran^1^, Niaz Chalabianloo^1^, Manik.Chhabra ^2,3,4^, Hugues Sampasa-Kanyinga ^5^, Flory Tsobo Muanda^1,2,3,4^

^1^Department of Physiology and Pharmacology, Western University, London, Ontario, Canada

^2^ICES Western, London, Ontario, Canada

^3^Department of Epidemiology & Biostatistics, Western University, London, Ontario, Canada

^4^Lawson Health Research Institute, London Health Sciences Centre, London, Ontario, Canada

^5^Healthy Active Living and Obesity Research Group, Children's Hospital of Eastern Ontario Research Institute, Ottawa, Ontario, Canada

**Corresponding Author**: Dr. Flory T Muanda, Department of Physiology and Pharmacology, Medical Sciences Building, 1151 Richmond St, Room 287, London, Ontario, Canada N6A 5C1 Tel: 519-661-2111-extension 84885,

Email: [fmuandat@uwo.ca](mailto:fmuandat@uwo.ca)

**Running title**: **Fluoroquinolones and the risk of panic attacks**

**Table S1: Preferred Reporting Items for Systematic Reviews and Meta-Analyses (PRISMA) 2020 for Abstracts Checklist**

| **Section and Topic** | **Item #** | **Checklist item** | **Reported (Yes/No)** |
| --- | --- | --- | --- |
| **TITLE** | | |  |
| Title | 1 | Identify the report as a systematic review. | Yes |
| **BACKGROUND** | | |  |
| Objectives | 2 | Provide an explicit statement of the main objective(s) or question(s) the review addresses. | Yes |
| **METHODS** | | |  |
| Eligibility criteria | 3 | Specify the inclusion and exclusion criteria for the review. | Yes |
| Information sources | 4 | Specify the information sources (e.g. databases, registers) used to identify studies and the date when each was last searched. | Yes |
| Risk of bias | 5 | Specify the methods used to assess risk of bias in the included studies. | Yes |
| Synthesis of results | 6 | Specify the methods used to present and synthesise results. | Yes |
| **RESULTS** | | |  |
| Included studies | 7 | Give the total number of included studies and participants and summarise relevant characteristics of studies. | Yes |
| Synthesis of results | 8 | Present results for main outcomes, preferably indicating the number of included studies and participants for each. If meta-analysis was done, report the summary estimate and confidence/credible interval. If comparing groups, indicate the direction of the effect (i.e. which group is favoured). | Yes |
| **DISCUSSION** | | |  |
| Limitations of evidence | 9 | Provide a brief summary of the limitations of the evidence included in the review (e.g. study risk of bias, inconsistency and imprecision). | Yes |
| Interpretation | 10 | Provide a general interpretation of the results and important implications. | Yes |
| **OTHER** | | |  |
| Funding | 11 | Specify the primary source of funding for the review. | Yes |
| Registration | 12 | Provide the register name and registration number. | Yes |

*From:*  Page MJ, McKenzie JE, Bossuyt PM, Boutron I, Hoffmann TC, Mulrow CD, et al. The PRISMA 2020 statement: an updated guideline for reporting systematic reviews. BMJ 2021;372:n71. doi: 10.1136/bmj.n71. This work is licensed under CC BY 4.0. To view a copy of this license, visit <https://creativecommons.org/licenses/by/4.0/>

**Table S2: Preferred Reporting Items for Systematic Reviews and Meta-Analyses (PRISMA) 2020 Checklist**

| **Section and Topic** | **Item #** | **Checklist item** | **Location where item is reported** |
| --- | --- | --- | --- |
| **TITLE** | | |  |
| Title | 1 | Identify the report as a systematic review. | Page 1 |
| **ABSTRACT** | | |  |
| Abstract | 2 | See the PRISMA 2020 for Abstracts checklist. | Page 2 |
| **INTRODUCTION** | | |  |
| Rationale | 3 | Describe the rationale for the review in the context of existing knowledge. | Page 4 |
| Objectives | 4 | Provide an explicit statement of the objective(s) or question(s) the review addresses. | Page 4 |
| **METHODS** | | |  |
| Eligibility criteria | 5 | Specify the inclusion and exclusion criteria for the review and how studies were grouped for the syntheses. | Page 5 |
| Information sources | 6 | Specify all databases, registers, websites, organisations, reference lists and other sources searched or consulted to identify studies. Specify the date when each source was last searched or consulted. | Page 5 |
| Search strategy | 7 | Present the full search strategies for all databases, registers and websites, including any filters and limits used. | Supplementary Material Table S3, Supplementary Material Table S4 |
| Selection process | 8 | Specify the methods used to decide whether a study met the inclusion criteria of the review, including how many reviewers screened each record and each report retrieved, whether they worked independently, and if applicable, details of automation tools used in the process. | Page 6 |
| Data collection process | 9 | Specify the methods used to collect data from reports, including how many reviewers collected data from each report, whether they worked independently, any processes for obtaining or confirming data from study investigators, and if applicable, details of automation tools used in the process. | Page 6 |
| Data items | 10a | List and define all outcomes for which data were sought. Specify whether all results that were compatible with each outcome domain in each study were sought (e.g. for all measures, time points, analyses), and if not, the methods used to decide which results to collect. | Page 6 |
|  | 10b | List and define all other variables for which data were sought (e.g. participant and intervention characteristics, funding sources). Describe any assumptions made about any missing or unclear information. | Page 6 |
| Study risk of bias assessment | 11 | Specify the methods used to assess risk of bias in the included studies, including details of the tool(s) used, how many reviewers assessed each study and whether they worked independently, and if applicable, details of automation tools used in the process. | Page 6-7 |
| Effect measures | 12 | Specify for each outcome the effect measure(s) (e.g. risk ratio, mean difference) used in the synthesis or presentation of results. | Page 7 |
| Synthesis methods | 13a | Describe the processes used to decide which studies were eligible for each synthesis (e.g. tabulating the study intervention characteristics and comparing against the planned groups for each synthesis (item #5)). | Page 6 |
|  | 13b | Describe any methods required to prepare the data for presentation or synthesis, such as handling of missing summary statistics, or data conversions. | Page 7 |
|  | 13c | Describe any methods used to tabulate or visually display results of individual studies and syntheses. | Fig. 1 |
|  | 13d | Describe any methods used to synthesize results and provide a rationale for the choice(s). If meta-analysis was performed, describe the model(s), method(s) to identify the presence and extent of statistical heterogeneity, and software package(s) used. | Page 7 |
|  | 13e | Describe any methods used to explore possible causes of heterogeneity among study results (e.g. subgroup analysis, meta-regression). | Page 7 |
|  | 13f | Describe any sensitivity analyses conducted to assess robustness of the synthesized results. | Page 7 |
| Reporting bias assessment | 14 | Describe any methods used to assess risk of bias due to missing results in a synthesis (arising from reporting biases). | Page 7 |
| Certainty assessment | 15 | Describe any methods used to assess certainty (or confidence) in the body of evidence for an outcome. | Page 7 |
| **RESULTS** | | |  |
| Study selection | 16a | Describe the results of the search and selection process, from the number of records identified in the search to the number of studies included in the review, ideally using a flow diagram. | Page 10 |
|  | 16b | Cite studies that might appear to meet the inclusion criteria, but which were excluded, and explain why they were excluded. | Page 10 |
| Study characteristics | 17 | Cite each included study and present its characteristics. | Pages 10-12 |
| Risk of bias in studies | 18 | Present assessments of risk of bias for each included study. | Supplementary Material Table S3, Supplementary Material Table S4 |
| Results of individual studies | 19 | For all outcomes, present, for each study: (a) summary statistics for each group (where appropriate) and (b) an effect estimate and its precision (e.g. confidence/credible interval), ideally using structured tables or plots. | Pages 10-12 |
| Results of syntheses | 20a | For each synthesis, briefly summarise the characteristics and risk of bias among contributing studies. | Pages 10-12 |
|  | 20b | Present results of all statistical syntheses conducted. If meta-analysis was done, present for each the summary estimate and its precision (e.g. confidence/credible interval) and measures of statistical heterogeneity. If comparing groups, describe the direction of the effect. | Pages 10-12 |
|  | 20c | Present results of all investigations of possible causes of heterogeneity among study results. | Pages 10-12 |
|  | 20d | Present results of all sensitivity analyses conducted to assess the robustness of the synthesized results. | Pages 10-12 |
| Reporting biases | 21 | Present assessments of risk of bias due to missing results (arising from reporting biases) for each synthesis assessed. | Pages 10-12 |
| Certainty of evidence | 22 | Present assessments of certainty (or confidence) in the body of evidence for each outcome assessed. | Pages 10-12 |
| **DISCUSSION** | | |  |
| Discussion | 23a | Provide a general interpretation of the results in the context of other evidence. | Pages 16-17 |
|  | 23b | Discuss any limitations of the evidence included in the review. | Page 20 |
|  | 23c | Discuss any limitations of the review processes used. | Page 20 |
|  | 23d | Discuss implications of the results for practice, policy, and future research. | Page 21 |
| **OTHER INFORMATION** | | |  |
| Registration and protocol | 24a | Provide registration information for the review, including register name and registration number, or state that the review was not registered. | Page 5 |
|  | 24b | Indicate where the review protocol can be accessed, or state that a protocol was not prepared. | Page 5 |
|  | 24c | Describe and explain any amendments to information provided at registration or in the protocol. | Page 22 |
| Support | 25 | Describe sources of financial or non-financial support for the review, and the role of the funders or sponsors in the review. | Page 22 |
| Competing interests | 26 | Declare any competing interests of review authors. | Page 22 |
| Availability of data, code and other materials | 27 | Report which of the following are publicly available and where they can be found: template data collection forms; data extracted from included studies; data used for all analyses; analytic code; any other materials used in the review. | Page 5 |

*From:*  Page MJ, McKenzie JE, Bossuyt PM, Boutron I, Hoffmann TC, Mulrow CD, et al. The PRISMA 2020 statement: an updated guideline for reporting systematic reviews. BMJ 2021;372:n71. doi: 10.1136/bmj.n71. This work is licensed under CC BY 4.0. To view a copy of this license, visit <https://creativecommons.org/licenses/by/4.0/>

**Table S3: Search strategy using keywords and Medical Subject Headings (MeSH) terms for the database MEDLINE [Indexed from 1946 to May 28, 2025].** ^[[1]](#footnote-1)^

| **#** | **Query** | **Results** |
| --- | --- | --- |
| 1 | exp Fluoroquinolones/ad, ae, po, tu, to [Administration & Dosage, Adverse Effects, Poisoning, Therapeutic Use, Toxicity] | 17313 |
| 2 | (fluoroquinolon* or ciprofloxacin? or norfloxacin? or gatifloxacin? or gemifloxacin? or moxifloxacin? or norfloxacin? or ofloxacin? or levofloxacin?).tw. | 68945 |
| 3 | 1 or 2 | 73017 |
| 4 | exp Anxiety Disorders/de, et [Drug Effects, Etiology] | 6378 |
| 5 | (panic attack$ or panic disorder or anxiety).mp. [mp=title, book title, abstract, original title, name of substance word, subject heading word, floating sub-heading word, keyword heading word, organism supplementary concept word, protocol supplementary concept word, rare disease supplementary concept word, unique identifier, synonyms, population supplementary concept word, anatomy supplementary concept word] | 363212 |
| 6 | 4 or 5 | 365801 |
| 7 | 3 and 6 | 64 |

**Table S4: Search strategy using keywords and Medical Subject Headings (MeSH) terms for the database Embase [Indexed from 1947 to May 28, 2025].**

| **#** | **Query** | **Results** |
| --- | --- | --- |
| 1 | exp quinolone derivative/ae, ct, ad, cm, dt, to, tm [Adverse Drug Reaction, Clinical Trial, Drug Administration, Drug Comparison, Drug Therapy, Drug Toxicity, Unexpected Outcome of Drug Treatment] | 99482 |
| 2 | (fluoroquinolon* or ciprofloxacin? or norfloxacin? or gatifloxacin? or gemifloxacin? or moxifloxacin? or norfloxacin? or ofloxacin? or levofloxacin?).tw. | 97791 |
| 3 | 1 or 2 | 174430 |
| 4 | exp anxiety disorder/et, si [Etiology, Side Effect] | 21250 |
| 5 | (panic attack$ or panic disorder or anxiety).mp. [mp=title, abstract, heading word, drug trade name, original title, device manufacturer, drug manufacturer, device trade name, keyword heading word, floating subheading word, candidate term word] | 607842 |
| 6 | 4 or 5 | 615286 |
| 7 | 3 and 6 | 3247 |

**Table S5: The Reporting of a Disproportionality Analysis for Drug Safety Signal Detection Using Individual Case Safety Reports in PharmacoVigilance (READUS-PV) checklist for abstracts**

| **Section and topic** | **Item #** | **Checklist item** | **Location where item is reported** |
| --- | --- | --- | --- |
| Background | *1a* | *State the aim/rationale for performing the study.* | Page 2 |
|  | *1b* | *Specify the adverse event(s) and/or the drug(s) under study, when applicable.* | Page 2 |
|  | *1c* | *Specify the specific population or setting, when applicable.* | Page 2 |
| Methods | *2a* | *Identify the study as a “disproportionality analysis” and specify the type of data used.* | Page 2 |
|  | *2b* | *Specify the name of the database(s) used and the type of access.* | Page 2 |
|  | *2c* | *Specify the timeframe and geographical region, when applicable.* | Page 2 |
|  | *2d* | *Specify the disproportionality measure(s) used and their statistical significance threshold(s).* | Page 2 |
|  | *2e* | *Specify if a case-by-case analysis is performed.* | Page 2 |
| Results | *3* | *Report main findings including their precision (e.g., 95% confidence intervals), together with a short summary of the case-by-case analysis.* | Page 2 |
| Conclusion | *4a* | *Clearly report key conclusions.* | Page 2 |
|  | *4b* | *Acknowledge that the disproportionality analysis is a hypothesis generating or refinement approach.* | Page 2 |
|  | *4c* | *State the implications and clinical relevance of the findings.* | Page 2 |

**Table S6: The Reporting of a Disproportionality Analysis for Drug Safety Signal Detection Using Individual Case Safety Reports in PharmacoVigilance (READUS-PV) checklist**

| **Section and topic** | **Item #** | **Checklist item** | **Location where item is reported** |
| --- | --- | --- | --- |
| **Title** |  |  |  |
|  | *1a* | *If disproportionality analyses are a prominent component of the published study, the study should be identified as a “disproportionality analysis”. The type of data and name of the database(s) should be specified.* | Page 1 |
|  | *1b* | *Report the name of adverse event(s) and/or drug(s) under study, when applicable.* | Page 1 |
| **Introduction** |  |  |  |
| Background | *2a* | *Describe the drug(s) and its utilization, the nature of the adverse event(s) under study and its frequency, and the existing knowledge on the drug-event combination.* | Page 4 |
|  | *2b* | *Specify the rationale for performing the analysis, e.g., as part of routine pharmacovigilance, to investigate an overall safety profile, or to assess a pre-specified hypothesis.* | Page 4 |
|  | *2c* | *Explain why ICSR databases and disproportionality analysis are suitable to fill the knowledge gap.* | Page 4 |
| Objectives | *3* | *State specific objectives, identifying the adverse event(s), the drug(s), and the reference group, including any pre-specified hypothesis, if applicable.* | Page 4 |
| **Methods** |  |  |  |
| Study design | *4a* | *Identify the study (i.e., “disproportionality analysis”) and the type of data used (e.g., “individual case safety reports”).* | Pages 7-8 |
|  | *4b* | *Provide an outline of the entire study design, including primary and sensitivity analyses performed, and other designs such as case-by-case analysis or literature review.* | Pages 7-8 |
| Data description, access, and pre-processing | *5a* | *Specify the name of the database(s), the database(s) custodian, and the coverage. Specify the type/number of drugs included within the database and the thesaurus, taxonomies, or ontologies used for coding drugs and events.* | Pages 7-8 |
|  | *5b* | *Specify the extraction dates and describe and justify all choices used for data pre-processing, including any data transformation or exclusion, if appropriate.* | Page 8 |
| Variables definition | *6a* | *Describe the study population, including any restriction.* | Pages 7-8 |
|  | *6b* | *Describe the nature and the meaning of key variables assessed in the work.* | Pages 8-9 |
|  | *6c* | *Specify and justify any grouping of drugs or events. For drugs, specify and justify whether active ingredients/trade names/salts were considered and/or the selected role.* | Page 9 |
|  | *6d* | *Describe any additional data source used, the type of data, and how they interact with ICSRs.* | Page 9 |
| Statistical methods | *7a* | *Present any descriptive analysis performed, specifying variables investigated, statistical tests, and significance thresholds.* | Pages 9-10 |
|  | *7b* | *Describe the measure(s) selected for the disproportionality analysis including any threshold used to identify signals of disproportionate reporting. Explain the reason for this choice if applicable.* | Pages 9-10 |
|  | *7c* | *Clearly describe any sensitivity analysis and any tool to control confounding, including any restriction, subgroup, stratification, adjustment, or interaction.* | Pages 9-10 |
|  | *7d* | *Specify the variables and methods used for the case-by-case analysis, including any algorithm or criteria used to assess causality, if performed.* | Pages 9-10 |
|  | *7e* | *Specify any statistical methods used for other data sources.* | Pages 9-10 |
| **Results** |  |  |  |
| Participants | *8a* | *Specify the number of individual case safety reports included at each stage, including reasons for exclusion.* | Page 13 |
|  | *8b* | *Provide key demographic and clinical characteristics of cases, if possible comparing cases with any appropriate reference group.* | Pages 13-14 |
| Disproportionality analysis | *9* | *Present all results including confidence intervals. Present also results of sensitivity analyses, if performed.* | Pages 13-15 |
| Case-by-case analysis | *10* | *Present the case-by-case analysis of key variables. Present the causality assessment, if applicable.* | Pages 14-16 |
| **Discussion** |  |  |  |
| Key results | *11* | *Discuss key results with reference to study objectives and contextualize them within the current literature and other consulted sources. Clearly discriminate between expected reactions and emerging safety signals.* | Pages 17-18 |
| External validity | *12a* | *Discuss the external validity of the results to the general population.* | Page 18 |
|  | *12b* | *Discuss the potential relevance of results in clinical practice* | Page 18 |
|  | *12c* | *Propose further study designs if applicable* | Page 21 |
| Limitations | *13* | *Present general limitations, making clear that disproportionality analysis alone cannot prove causation or measure incidence, and specific limitations, including confounding and reporting bias and efforts to mitigate them.* | Pages 20-21 |
| **Declarations** |  |  |  |
|  | *14a* | *Provide the source of funding/sponsorship and the role of the funders/sponsors for the present study and for any original study on which the present article is based.* | Page 22 |
|  | *14b* | *Clearly identify potential commercial and intellectual conflicts of interest (e.g., link to any drug/event investigated, whether financial, legal action, or software used).* | Page 22 |
|  | *14c* | *Declare any institutional approval needed or granted in the investigation.* | Page 22 |
|  | *14d* | *Include a statement on data availability, code availability (including the version of the statistical software used), and protocol registration.* | Pages 8-9 |

**Table S7: Comparison of labelled indications for ciprofloxacin, levofloxacin, moxifloxacin, azithromycin, and trimethoprim-sulfamethoxazole according to UpToDate.**

| **Drug** | **Labelled Indications** |
| --- | --- |
| Ciprofloxacin | Urinary tract infections, acute uncomplicated cystitis in females, chronic bacterial prostatitis, bone and joint infections, complicated intra-abdominal infections (with metronidazole), infectious diarrhea, typhoid fever, hospital-acquired pneumonia |
| Levofloxacin | Community-acquired pneumonia, nosocomial pneumonia, chronic obstructive pulmonary disease, acute bacterial rhinosinusitis, prostatitis, urinary tract infections, pyelonephritis, skin and skin structure infections, anthrax, plague |
| Moxifloxacin | Community-acquired pneumonia, acute bacterial rhinosinusitis, skin and skin structure infections, complicated intra-abdominal infections, plague |
| Azithromycin | Chancroid, chronic obstructive pulmonary disease exacerbation, mycobacterium avium complex prevention, otitis media, pelvic inflammatory disease, community-acquired pneumonia, skin and skin structure infections, streptococcal pharyngitis |
| Trimethoprim-sulfamethoxazole | Urinary tract infections, otitis media, chronic obstructive pulmonary disease exacerbations, pneumocystis pneumonia, traveller’s diarrhea, shigellosis |

**Reference: UpToDate Lexidrug. Wolters Kluwer; 2026. Accessed February 11, 2026**

**Table S8: Summary of clinical trials reporting panic attacks or acute anxiety as an outcome linked to fluoroquinolones (FQs) included in the systematic review.**

| **Citation** | **Location** | **Population** | **Primary Objective** | **Type of fluoroquinolones** | **Comparator** | **Outcome** | **Quality** |
| --- | --- | --- | --- | --- | --- | --- | --- |
| Medeiros 2002 ^1^ | Multicenter in Brazil | N=5044 (45.4% male) | To evaluate the efficacy of gatifloxacin in the treatment of community-acquired respiratory tract infections. | Gatifloxacin (400 mg), QD,  7-14 days | N/A | CNS alterations, including anxiety:  23/5044  (0.5%) | ROB2: High |
| Periti et al. 1998 ^2^ | Multicenter in Italy | N=153  (68.6% male) | To determine the efficacy and safety of IV Ciprofloxacin for acute bacterial pneumonia. | Ciprofloxacin (400 mg), IV, BID | N/A | Anxiety episode: 1/153 (0.7%) | ROB2: High |
| Petitpretz et al. 2007 ^3^ | Multicenter in France, Germany, Tunisia, Belgium, Austria and Turkey | N=689  (81.4% male) | To confirm the non-inferiority of levofloxacin for the treatment of AECOB. | Oral Levofloxacin (500 mg), QD, 10 days  (N=340) | Cefuroxime (250 mg), BID, 10 days  (N=349) | Panic Attack  Levofloxacin:  6/340  (1.8%)  Cefuroxime:  2/349  (0.6%) † | ROB2:  Some concerns |
| Tack et al. 1995 ^4^ | Multicenter in the United States of America | N=76  (46.1% male) | To administer clinafloxacin to patients who may benefit from the medication | Clinafloxacin (200 mg), IV or orally every 12 hours, 7 to 14 days  May have been increased to 300 mg or 400 mg every 12 hours for more resistant pathogens and/or larger patients | N/A | Anxiety: 1/76  (1.3%) | ROB2: High |

Abbreviations: QD = once daily; CNS- central nervous system; IV- intravenous; BID = twice daily; AECOB- acute exacerbation of chronic obstructive bronchitis; CI = confidence interval

† Risk ratio = 3.08 (95% CI [0.63 to 15.15]).

**Table S9: Risk of bias analysis of clinical trials included in the systematic review with the use of the Cochrane risk-of-bias tool (Version 2).**

| **Citation** | **Randomization Process** | **Deviations from intended intervention** | **Missing Outcome Data** | **Measurement of outcome** | **Selective reporting** | **Overall Risk of Bias** |
| --- | --- | --- | --- | --- | --- | --- |
| Medeiros 2002 | High | High | Some Concerns | Some Concerns | Low | High |
| Periti et al. 1998 | High | High | High | Some Concerns | Low | High |
| Petitpretz et al. 2007 | Low | Some Concerns | Low | Some Concerns | Low | Some Concerns |
| Tack et al. 1999 | High | High | Some Concerns | High | Some Concerns | High |

**Table S10: Summary of case reports reporting panic attacks or acute anxiety as an outcome linked to fluoroquinolones (FQs) included in the systematic review.**

| **Citation** | **Population** | **Indication** | **fluoroquinolones Administered** | **Time to Event** | **Treatment** | **Time to Relief** | **Quality** |
| --- | --- | --- | --- | --- | --- | --- | --- |
| Abusafiyah and Soulen 2023 ^5^ | 52-year-old male | Indwelling common bile duct stent and a history of cholangitis | Moxifloxacin prophylaxis (400 mg), QD, oral, 3 days before percutaneous ablation of a solitary liver metastasis | 2 days to acute anxiety | Stopped moxifloxacin; discharged with amoxicillin (500 mg) /clavulanate (125 mg) BID and metronidazole (500 mg) BID | Next few days | Naranjo Score: 3 |
| Hall et al. 2003  (Case 1) ^6^ | 25-year-old woman | PID | Ofloxacin (400 mg), BID | 12 hours to acute anxiety | N/A | 1 day after full ofloxacin treatment | Naranjo Score: 5 |
| Hall et al. 2003  (Case 2) | 32-year-old woman | PID | Ofloxacin (400 mg), BID | Few hours after first dose to panic attack | Discontinued ofloxacin; started on doxycycline | 2 days | Naranjo Score: 6 |
| Kandasamy and Srinath 2012  (Case 1) ^7^ | 30-year-old male | Lower respiratory tract infection | Levofloxacin (500 mg), QD, 5 days | 2 days to acute anxiety | Prescribed a different class of antibiotic | 2 days for reduction in anxiety and then remission | Naranjo Score: 6 |
| Kandasamy and Srinath 2012  (Case 2) | 30-year-old woman | Lower respiratory tract infection | Levofloxacin (500 mg), QD | Anxiety after first dose | Stopped levofloxacin | 1 day | Naranjo Score: 6 |
| Kandasamy and Srinath 2012  (Case 3) | 32-year-old man | Acute lower respiratory tract infection | Levofloxacin (750 mg) tablets | 4 to 6 hours to acute non-specific anxiety | Stopped levofloxacin | 1 day | Naranjo Score: 7 |
| Maharani et al. 2019 ^8^ | 19-year-old male | Acute sinusitis | Levofloxacin (500 mg), QD, 5 days | Half-an-hour to acute anxiety after intake of first dose | Switched to amoxicillin and clavulanic acid with continued paracetamol and naphazoline decongestant | N/A | Naranjo Score: 6 |
| Mazhar et al. 2016 ^9^ | 40-year-old male | Acute bronchitis | Moxifloxacin (400 mg), QD, 5 days | 72 hours to acute anxiety | Discontinue moxifloxacin and start azithromycin | 12 hours | Naranjo Score: 5 |
| Muradian and Khan 2019 ^10^ | 23-year-old woman | Bilateral sub-mandibular abscesses | Levofloxacin | 3 days to acute anxiety | Discontinued levofloxacin; transitioned to ceftriaxone | 3 days | Naranjo Score: 7 |
| Müssig et al. 2009 ^11^ | 73-year-old woman | Acute febrile  upper airway infection | Moxifloxacin (400 mg), QD | In the following days, acute anxiety was present | Discontinued moxifloxacin; initiated IV saline treatment | 8 days | Naranjo Score: 7 |
| Strauchman and Morningstar 2012 ^12^ | 38-year-old woman | Sinusitis | Moxifloxacin HCl (Avelox) (500 mg), QD, 14 days | Panic Attacks for the 2-week period | Prescribed multivitamin, broad-spectrum amino acid supplement,  omega-3 fatty acid supplement, magnesium,  and malic acid. Also, given liothyronine sodium (Cytomel) | N/A | Naranjo Score: 7 |

Abbreviations: QD = once daily; BID = twice daily; PID- pelvic inflammatory disease; IV- intravenous.

**Table S11: The Naranjo adverse drug reaction probability scale of case studies included in the systematic review**

| **Naranjo Item** | **Abusafiyah and Soulen 20232** | **Hall et al. 2003**  **(Case 1)** | **Hall et al. 2003**  **(Case 2)** | **Kandasamy and Srinath 2012**  **(Case 1)** | **Kandasamy and Srinath 2012**  **(Case 2)** | **Kandasamy and Srinath 2012**  **(Case 3)** | **Maharani et al. 2019** | **Mazhar et al. 2016** | **Muradian and Khan 2019** | **Müssig et al. 2009** | **Strauchman and Morningstar 2012** |
| --- | --- | --- | --- | --- | --- | --- | --- | --- | --- | --- | --- |
| Are there previous conclusive reports on this reaction? | 1 | 1 | 1 | 1 | 1 | 1 | 1 | 1 | 1 | 1 | 1 |
| Did adverse event appear after the suspected drug was given? | 2 | 2 | 2 | 2 | 2 | 2 | 2 | 2 | 2 | 2 | 2 |
| Did the adverse reaction improve when the drug was discontinued or a specific antagonist was given? | 1 | 0 | 1 | 1 | 1 | 1 | 1 | 1 | 1 | 1 | 1 |
| Did the adverse reaction appear when the drug was readministered? | 0 | 0 | 0 | 0 | 0 | 0 | 0 | 0 | 0 | 0 | 2 |
| Are there alternative causes that could have caused the reaction? | -1 | 2 | 2 | 2 | 2 | 2 | 2 | 0 | 2 | 2 | -1 |
| Did the reaction reappear when a placebo was given? | 0 | 0 | 0 | 0 | 0 | 0 | 0 | 0 | 0 | 0 | 0 |
| Was the drug detected in any body fluid in toxic concentrations? | 0 | 0 | 0 | 0 | 0 | 0 | 0 | 0 | 0 | 0 | 0 |
| Was the reaction more severe when the dose was increased, or less severe when the dose was decreased? | 0 | 0 | 0 | 0 | 0 | 1 | 0 | 0 | 0 | 0 | 0 |
| Did the patient have a similar reaction to the same or similar drugs in any previous exposure? | 0 | 0 | 0 | 0 | 0 | 0 | 0 | 0 | 0 | 0 | 1 |
| Was the adverse event confirmed by any objective evidence? | 0 | 0 | 0 | 0 | 0 | 0 | 0 | 1 | 1 | 1 | 1 |
| **Score** | 3 | 5 | 6 | 6 | 6 | 7 | 6 | 5 | 7 | 7 | 7 |

Case reports were assigned a Naranjo score from -4 to +13. Scores correspond to one of four quality levels: definite (scores 9 to 13), probable (scores 5 to 8), possible (scores 2 to 4), and doubtful (scores less than 2)


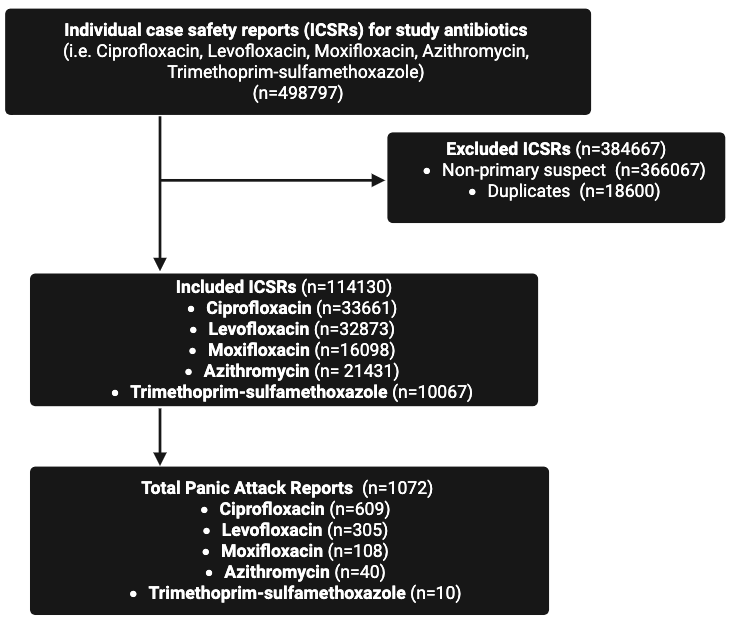


**Figure S1: Flow diagram of identifying panic attack reports from the FDA Adverse Event Reporting System (FAERS) database for the active-comparator restricted disproportionality analysis (ACR-DA).**

Flow diagram illustrating the processing of individual case safety reports (ICSRs) for study antibiotics (ciprofloxacin, levofloxacin, moxifloxacin, azithromycin, trimethoprim-sulfamethoxazole) for the active-comparator restricted disproportionality analysis (ACR-DA) from the FDA Adverse Event Reporting System (FAERS) database. ICSRs were submitted by consumers, healthcare professionals, or pharmaceutical companies. The non-primary suspect and duplicate ICSRs were excluded. Using the Medical Dictionary for Regulatory Activities (MedDRA) term "panic attacks," panic attack reports for study antibiotics were identified.

**References:**

1. Medeiros EAS. Treatment of adults with community-acquired respiratory tract infections: results of a multicentric clinical trial with gatifloxacin. *Brazilian Journal of Infectious Diseases*. 2002;6(4):149-156. doi:10.1590/S1413-86702002000400001

2. Periti P, Mazzei T, Elena Curti M. Efficacy and safety of high dose intravenous ciprofloxacin in the treatment of bacterial pneumonia. *Int J Antimicrob Agents*. 1998;10(3):215-222. doi:10.1016/S0924-8579(98)00039-9

3. Petitpretz P, Choné C, Trémolières F. Levofloxacin 500 mg once daily versus cefuroxime 250 mg twice daily in patients with acute exacerbations of chronic obstructive bronchitis: clinical efficacy and exacerbation-free interval. *Int J Antimicrob Agents*. 2007;30(1):52-59. doi:10.1016/J.IJANTIMICAG.2006.11.033

4. Tack KJ, McGuire NM, Eiseman IA. Initial Clinical Experience with Clinafloxacin in the Treatment of Serious Infections. *Drugs*. 1995;49(2):488-491. doi:10.2165/00003495-199500492-00145/METRICS

5. Abusafiyah N, Soulen MC. Driving Patients Crazy: Acute Neuropsychiatric Toxicities of Commonly Prescribed Antibiotics in IR. *Journal of Vascular and Interventional Radiology*. 2023;34(10):1722-1724. doi:10.1016/j.jvir.2023.06.010

6. Hall CE, Keegan H, Rogstad KE. Psychiatric side effects of ofloxacin used in the treatment of pelvic inflammatory disease. *Int J STD AIDS*. 2003;14(9):636-637. doi:10.1258/095646203322301121

7. Kandasamy A, Srinath D, Kandasamy A, Srinath D. Levofloxacin-induced acute anxiety and insomnia. *J Neurosci Rural Pract*. 2011;3(2):212-214. doi:10.4103/0976-3147.98256

8. Maharani B, Jafrin A, Bai K, Sivagnanam G. Levofloxacin-induced tactile hallucination and acute anxiety reaction. *Indian J Pharmacol*. 2019;51(2):123-125. doi:10.4103/IJP.IJP_291_17

9. Mazhar F, Akram S, Haider N. Moxifloxacin-induced acute psychosis: A case report with literature review. *J Res Pharm Pract*. 2016;5(4):294. doi:10.4103/2279-042X.192457

10. Muradian M, Khan S, Muradian M, Khan S. Levofloxacin-induced Psychosis in a Young Healthy Patient. *Cureus*. 2019;11(11). doi:10.7759/CUREUS.6217

11. Müssig K, Schnauder G, ’Mörike K. Severe and symptomatic hyponatraemia after moxifloxacin intake. *Neth J Med*. 2009;67(5):197.

12. Strauchman M, Morningstar MW. Fluoroquinolone Toxicity Symptoms in a Patient Presenting with Low Back Pain. *Clinics and Practice 2012, Vol 2,*. 2012;2(4):e87. doi:10.4081/CP.2012.E87

1. Clinical indications was obtained from: Hooper et al. Fluoroquinolones. UpToDate, Version 2025. Available from: https://www.uptodate.com/contents/fluoroquinolones. Accessed May 28, 2025. [↑](#footnote-ref-1)
